# Comparing guided and unguided forest bathing for graduate student stress management: a pilot study

**DOI:** 10.3389/fpsyg.2026.1759571

**Published:** 2026-04-09

**Authors:** Zhiyong Zhang, Yue Gao, Xiaoyu Huang, Bing Ye

**Affiliations:** Research Institute of Forestry Policy and Information, Chinese Academy of Forestry, Beijing, China

**Keywords:** cost-effectiveness, emotion regulation, forest bathing, graduate students, nature therapy, psychological wellbeing, restorative environment

## Abstract

**Background:**

The escalating mental health crisis among graduate students has become a pressing public health concern, necessitating the development of effective, accessible, and low-cost stress management strategies. While exposure to green spaces is known to benefit psychological well-being, the differential efficacy of structured versus unstructured forest activities remains understudied.

**Methods:**

This investigation evaluated the emotion regulation benefits of short-term forest healing experiences among graduate students in Beijing. The study compared the effects of forest versus urban environments and compared therapist-guided versus unguided modalities.

**Results:**

Results indicated that: (1) Brief outdoor activities significantly reduced negative emotions (tension, depression, anger, fatigue, confusion) across all groups; (2) Compared with the urban environment, the forest environment demonstrated greater efficacy in regulating emotions, supported by superior environmental quality (e.g., higher negative air ions, lower noise) ; and (3) Notably, the presence of a forest therapist did not yield statistically superior benefits compared to unguided forest exposure.

**Conclusion:**

These findings suggest that self-guided forest bathing serves as a sufficient, cost-effective, and easily accessible intervention for daily stress reduction. Consequently, promoting unguided nature exposure offers a scalable public health solution to mitigate the mental health burden in high-stress academic populations.

## 1 Introduction

Emotion is a term for an affective state, encompassing specific positive and negative affective states, such as anger, amusement, and sadness (Gross, 2015). Positive emotions can enhance the decision-making ability of people, help them formulate correct action plans, increase work efficiency, and better handle conflicts and struggles. In contrast, negative emotions can impair the cognitive function and behavior of people, especially if they arise at inappropriate times (Gross and Jazaieri, 2014). Recent findings suggest that emotions involve coupled changes in various domains such as subjective experience, behavior, and physiology (Feiler et al., 2023). Emotional valence not only influences individuals’ perception and attitudes toward events but also triggers physiological changes in the body. Prolonged exposure to negative emotional states increases the risk of developing psychopathological symptoms. Thus, emotion regulation is crucial for both physical and psychological health as a process through which individuals regulate their own affective states (Gatto et al., 2022).

The postgraduate phase constitutes a critical transitional period in academic progression. Building upon foundational undergraduate training, postgraduates demonstrate significantly increased capacities for independent inquiry and self-regulated learning. However, this population simultaneously faces complex expectations from society, family, and themselves, while navigating multiple stressors including, but not limited to, academic demands, career uncertainties, socioeconomic factors, and developmental transitions (Schut et al., 2020). Recent international epidemiological studies highlight that the mental health crisis among graduate students has become a global challenge. A landmark survey by Evans et al. (2018) spanning 26 countries revealed that graduate students are more than six times as likely to experience depression and anxiety as compared to the general population (Evans et al., 2018). This alarming trend was further corroborated by a systematic review and meta-analysis, which reported that the pooled prevalence of clinically significant symptoms of depression and anxiety among PhD students was 24 and 17%, respectively (Satinsky et al., 2021). In China, this issue is exacerbated by the sustained and rapid expansion of postgraduate enrollment in recent years. According to the *2023 National Education Development Statistical Bulletin*, the total number of enrolled postgraduates reached 3.88 million in 2023, reflecting a year-on-year increase of 229,300 students (6.28%). These included 612,500 doctoral candidates and 3.27 million master’s candidates (Ministry of Education of the People’s Republic of China, 2024). The growing number of graduate student populations has, to some extent, heightened competition, contributing to a rise in mental health concerns. Consequently, a pressing practical need exists for stress alleviation and regulation of daily emotional distress.

Numerous studies have reported that exposure to green spaces can have multiple positive health effects, especially in terms of mental health (Kabisch et al., 2021; Wang et al., 2022; Queirolo et al., 2024; Yin et al., 2025; Zhang et al., 2025). Shin et al. conducted a randomized 3 × 3 crossover study involving university students exposed to various conditions. Psychological assessments revealed that forest therapy yielded significantly greater improvements in mood regulation and anxiety reduction compared with conventional daily activities (Shin et al., 2023). Subirana-Malaret et al. evaluated the emotional states of participants (*N* = 1142) from 35 countries following guided forest bathing walks. The results demonstrated that feelings of happiness and trust were rated extremely highly after participating in forest bathing activities (Subirana-Malaret et al., 2023). Another experiment conducted in Barcelona, Spain, demonstrated that forest therapy significantly enhanced positive emotions while reducing anxiety and negative mood (Muro et al., 2023). Eye-tracking studies further revealed that focusing visual attention on natural elements (as opposed to urban gray structures) significantly reduced psychological distress (Fleming et al., 2024). Current literature has established the salutary effects of forest-based activities on mood regulation. However, current literature has largely focused on comparing forest versus urban settings, with insufficient attention paid to the cost-effectiveness of intervention modalities. From the perspective of public health resource allocation, professional mental health resources are often scarce and costly. Therefore, verifying whether “unguided forest exposure” can achieve outcomes comparable to “therapist-guided forest therapy” is critical for designing cost-effective and scalable intervention policies (Buckley et al., 2019; Kondo et al., 2020). Elucidating this distinction is essential for determining the necessity of professional involvement in nature-based interventions.

Therefore, bridging the gap between environmental psychology and public health resource allocation, this study employed a cohort of graduate students in Beijing to examine the differential effects of forest versus urban environments, with a specific focus on evaluating whether therapist-guided sessions provide additional psychophysiological benefits compared to unguided forest exposure, thereby informing cost-effective resource allocation. We aimed to elucidate not only the acute psychophysiological responses across these conditions but also the potential added value of professional guidance. By determining whether unguided exposure can yield comparable benefits to therapist-led sessions, this study seeks to provide evidence-based recommendations for developing cost-effective, scalable, and accessible nature-based strategies to mitigate the growing mental health crisis in high-academic-stress populations.

## 2 Materials and methods

### 2.1 Participants

This study was designed as a preliminary pilot trial to investigate the feasibility and potential efficacy of forest healing interventions. Thirty graduate students were recruited through a bulletin at the Chinese Academy of Forestry. Regarding user availability and readiness, all candidates underwent a pre-screening process. Inclusion criteria required participants to be: (1) history of outdoor allergens; (2) diagnosis of severe stress or depression; (3) drug or alcohol abuse; and (4) available to commit to the full 3-h intervention without external distractions.

Prior to the study, a detailed briefing was provided, and written informed consent was obtained from each participant, ensuring their voluntary participation and the right to withdraw at any time. This study was conducted in accordance with the Declaration of Helsinki. Each participant was provided with coffee coupons worth CNY 200 as compensation for time commitment.

### 2.2 Study site and experimental conditions

**FIGURE 1:**
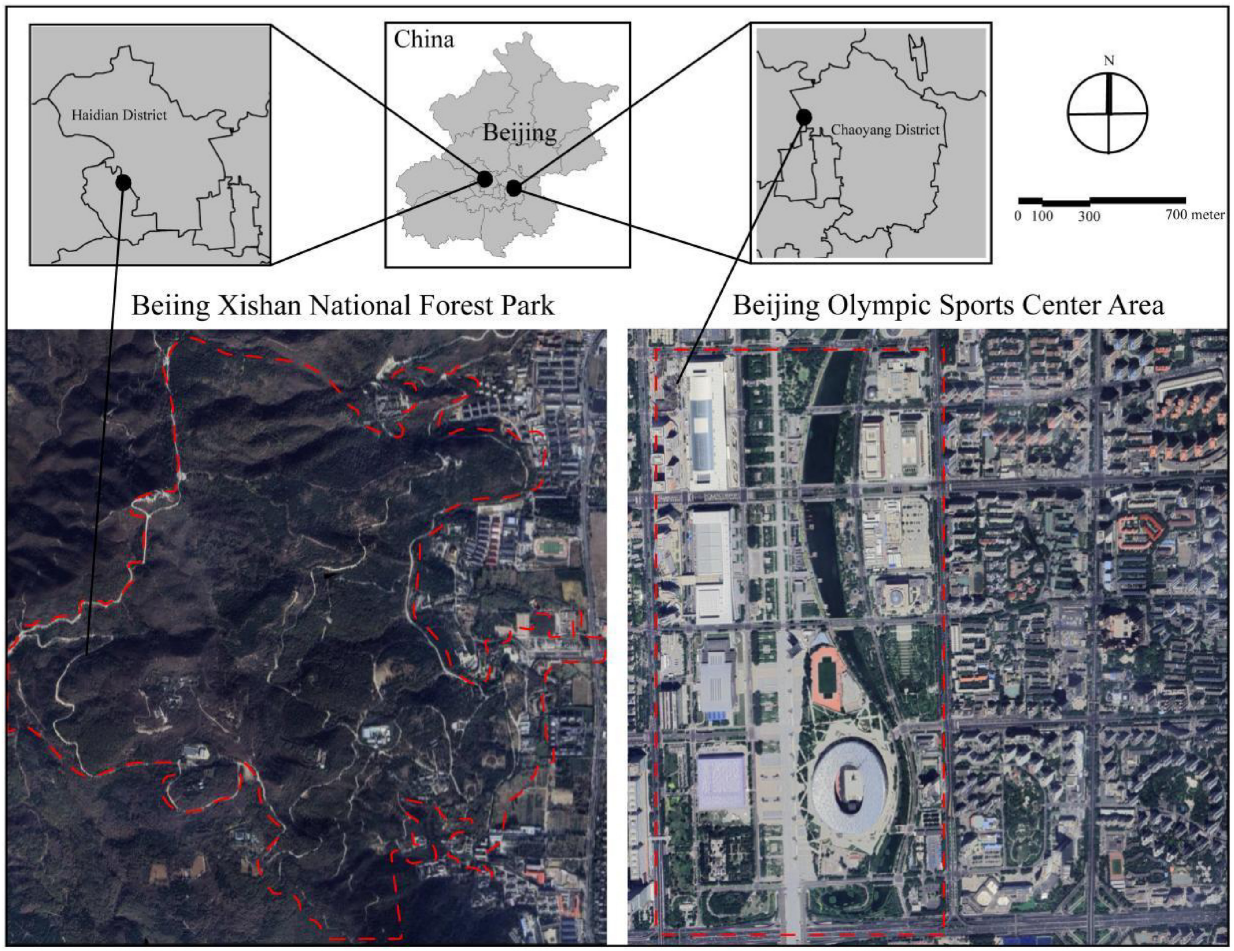
Location of the study site in Beijing, China.

The therapeutic interventions (sessions of therapist-guided forest healing and unguided forest exposure) were conducted at Beijing Xishan National Forest Park (39°58’18.17”N, 116°11’51.20”E), the proximal forested area to Beijing’s urban core (Figure 1a). The park is situated in a temperate monsoon climate zone, characterized by distinct seasonal variations. This experimental site represented a temperate deciduousate deciduousvariations. This experimenta% vegetation coverage dominated by *Pinus tabulaeformis*, *Platycladus orientalis*, *Cotinus coggygria*, *Robinia pseudoacacia*, and *Koelreuteria paniculata* (Yang et al., 2022), providing a representative landscape of North China’ s mountainous forests. The urban control site was set at the Beijing Olympic Sports Center Area (Figure 1b). This area had limited vegetation cover and consisted of high-density built environment with sports facilities (e.g., National Stadium: Bird’s Nest; National Aquatics Center: Water Cube), convention centers (China National Convention Center), and commercial areas (Xin’ao Shopping Center). Field experiments were conducted during the autumnal optimum (mid-October 2023), which is a temporal window characterized by peak foliar senescence, thermal comfort conditions, and atmospheric clarity. All trials were performed under stable weather conditions, ensuring that the participants had the best psychological experience.

### 2.3 Research design

A between-subjects randomized experimental design was employed to compare the effects of different forest healing modalities. To ensure methodological rigor and minimize selection bias, the 30 participants were randomly allocated into three groups (10 participants per group). Specifically, a random number sequence was generated using Microsoft Excel, and participants were allocated to the groups based on their enrollment numbers corresponding to the generated sequence, and a single-blind approach was applied during data analysis, where the primary analyst remained unaware of the group identities until the statistical procedures were completed. One group participated in a forest healing program guided by a forest therapist, and another group participated in a forest exposure program without a forest therapist. The two groups performed activities in Beijing Xishan National Forest Park. Meanwhile, the other group was involved in urban exposure program without a forest therapist in the Beijing Olympic Sports Center Area. All three groups performed their activities on the same day, with each session lasting 3 h (14:00–17:00), starting and ending simultaneously.

**FIGURE 2:**
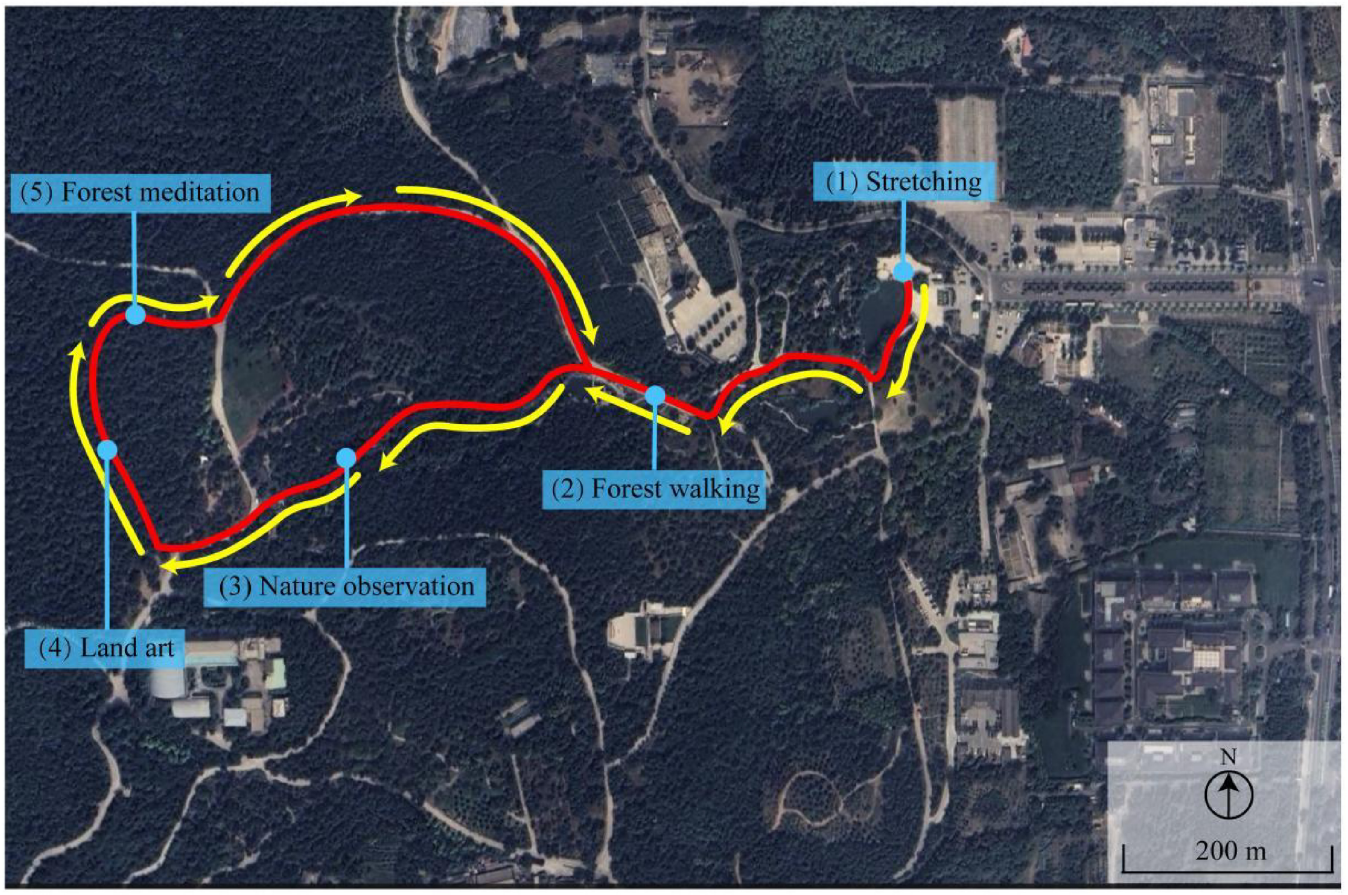
The activity routes of therapist-guided forest healing program.

The activities performed by the therapist-guided forest healing group were structured into five distinct phases to engage different sensory and cognitive pathways (Figure 2): (1) Stretching (20 min): Participants performed guided rhythmic stretching exercises focused on relaxing major muscle groups and regulating breathing to transition from a daily working state to a relaxed state. (2) Forest Walking (40 min): A slow-paced walk (< 2 km/h) was conducted. Participants were instructed to “walk mindfully, feeling the texture of the soil underfoot and synchronizing breath with steps.” (3) Nature Observation (30 min): Utilizing the “Five Senses” technique, the therapist guided participants to visually focus on the fractal patterns of leaves, listen to birdsong and wind, and smell the phytoncides, promoting sensory immersion. (4) Land Art (40 min): Participants were invited to collect fallen leaves, twigs, and stones to create a temporary art piece on the ground, facilitating non-verbal emotional expression. (5) Forest Meditation (20 min): The session concluded with a guided mindfulness meditation. Participants sat on bench, closing their eyes while the therapist provided verbal cues to “scan the body for tension and release it into the earth.” In contrast, the other two groups (unguided forest exposure and urban exposure) were primarily engaged in free walking without specific instructions or structured interactions.

Baseline demographic data, including sex, age, and sleep quality (rated on a 5-point scale: 1, excellent; 2, normal; 3, insufficient; 4, occasional insomnia; 5, chronic insomnia), were collected prior to the experiment. The emotional states were monitored both before and after the intervention. The restorative characteristics of forest and urban environments were evaluated simultaneously during the activities. Furthermore, synchronous real-time monitoring was implemented using portable environmental measurement devices throughout the activity periods to further characterize the differences in the two environments.

### 2.4 Measurements

Profile of Mood States (POMS) was used to evaluate the emotional states of participants. It was developed by McNair et al. It evaluates six mood states: tension-anxiety (T-A), depression-dejection (D), anger-hostility (A-H), fatigue (F), confusion (C), and vigor (V) (McNair et al., 1992). A higher score indicates greater intensity of each emotional state. The total mood disturbance (TMD) score was calculated using the following formula: (T-A) + (D) + (A-H) + (F) + (C)—(V). A high TMD score reflected a poor emotional state (Shin et al., 2023). The Chinese version of POMS, comprising 65 items, was used in this study. Each question was evaluated on a 5-point Likert scale (0 = “not at all” to 4 = “extremely”) through self-report (Kim et al., 2023).

The restoration environment scale was also used to evaluate the restoration characteristics of forest and urban environments. The restoration environment assessment originated from the Attention Restoration Theory (ART) proposed by Rachel and Stephen Kaplan in 1989. This theory holds that the restorative environments are characterized by four dimensions: being away (B-A), extent (E), fascination (Fa), and compatibility (Co) (Kaplan and Kaplan, 1989). In this study, we employed the Chinese version of the restorative environment scale, which was developed by Ye et al. (2010). The scale comprised 22 items rated on a 7-point Likert scale (1 = strongly disagree to 7 = strongly agree). Higher scores indicated stronger manifestation of these characteristics.

The portable instruments were employed to quantitatively characterize environmental conditions at two sites, with synchronous measurements of the following key parameters: (1) Meteorological variables (temperature, relative humidity, and wind speed) were recorded using a handheld weather station (NK5500, Kestrel, United States). (2) Negative air ion concentration was measured using an air ion counter (WST-05A, Wostron Tech, China). (3) Noise levels were quantified using a sound level meter (HY128-2, Hunan Shengyi Instrument Co., China). (4) Particulate matter (PM_2_._5_ and PM_10_) was monitored using a handheld dust monitor (DustMate, Turnkey, United Kingdom).

### 2.5 Statistical analysis

Descriptive statistics included means, standard deviation (SD), and percentages to represent outcome variables. Statistical analyses were performed using IBM SPSS Statistics 21.0 (IBM SPSS software, NY, United States). First, one-way ANOVA was employed to ensure there were no significant baseline differences between the three experimental groups. Subsequently, one-way Analysis of Covariance (ANCOVA) was conducted to compare post-intervention scores, with baseline scores as covariates. The normality of the data was assessed using the Shapiro-Wilk test, and homogeneity of variance was verified via Levene’s test Correlation analysis was used to indicate the interrelationships among various factors. A *P*-< 0.05 indicated a statistically significant difference.

## 3 Results

### 3.1 Demographic information

The study excluded incomplete questionnaires and those with predominantly identical responses across items. After data screening, the valid samples comprised eight questionnaires for the therapist-guided forest healing group, nine questionnaires for the unguided forest exposure group, and nine questionnaires for the urban exposure group. The demographic characteristics of participants in each group are presented in Table 1. The sample characteristics were as follows: therapist-guided forest healing group (*n* = 8) included participants aged 23–28 years (mean ± SD: 25.00 ± 1.69), predominantly female (77.78%), with sleep quality scores between normal and insufficient ranges (2.63 ± 1.30); unguided forest exposure group (*n* = 9) included participants aged 21–27 years (23.33 ± 1.87), predominantly female (70.00%), with sleep quality scores between normal and insufficient ranges (2.11 ± 1.27); and urban exposure group (*n* = 9) included participants aged 21–28 years (24.56 ± 2.30), predominantly female (70.00%), with sleep quality scores between normal and insufficient ranges (2.44 ± 1.51). No statistically significant intergroup differences were observed in baseline demographic variables (*P* > 0.05).

**TABLE 1:** The demographic characteristics of participants in each group.

No	Therapist-guided forest healing			Unguided forest exposure			Urban exposure		
	Sex	Age	Sleep quality	Sex	Age	Sleep quality	Sex	Age	Sleep quality
1	Female	25	1	Female	21	1	Female	24	1
2	Female	25	4	Female	22	1	Female	25	1
3	Female	23	3	Male	22	1	Male	26	4
4	Female	24	4	Female	22	2	Female	27	4
5	Female	28	4	Male	24	1	Female	21	4
6	Male	26	2	Female	27	2	Male	28	2
7	Male	26	2	Male	25	3	Female	22	1
8	Female	23	1	Female	24	4	Male	25	4
9	/	/	/	Female	23	4	Female	23	1
N (%) or Mean ± SD	Male: 25.00% Female: 75.00%	25.00 ± 1.69	2.63 ± 1.30	Male: 30.00% Female: 70.00%	23.33 ± 1.87	2.11 ± 1.27	Male: 30.00% Female: 70.00%	24.56 ± 2.30	2.44 ± 1.51

The statistical analysis of pre-experiment POMS scores revealed no significant differences among the three groups across six emotional dimensions: tension-anxiety, depression-dejection, anger-hostility, fatigue, confusion, vigor, and TMD (*P* > 0.05; Table 2). The homogeneous distribution of baseline emotional states across groups confirmed the scientific rigor of the randomization process. This baseline validation is crucial, as it ensures that the subsequent psychological improvements are attributable to the environmental and modal differences rather than initial emotional variances, thereby reinforcing the statistical reliability of the intervention results.

**TABLE 2:** The differences of POMS scores before activity among the groups.

Mood states	Therapist-guided forest healing	Unguided forest exposure	Urban exposure
Tension-anxiety	7.50 ± 3.07	8.00 ± 3.39	7.67 ± 1.73
Depression-dejection	7.38 ± 4.27	7.33 ± 6.10	7.33 ± 4.82
Anger-hostility	5.75 ± 4.30	4.44 ± 2.92	4.11 ± 3.10
Fatigue	7.75 ± 2.82	7.56 ± 3.94	6.11 ± 2.62
Confusion	8.38 ± 4.37	9.56 ± 3.94	9.22 ± 3.03
Vigor	17.38 ± 2.88	17.22 ± 4.89	17.89 ± 4.59
TMD	19.38 ± 15.07	19.67 ± 15.36	16.56 ± 14.03

### 3.2 Environmental conditions

#### 3.2.1 Monitoring index

Comparative environmental monitoring data between forest and urban environments displayed statistically significant differences (*P* < 0.01 except temperature) across multiple monitoring indices (Table 3). Specifically, forest environment monitoring data demonstrated lower temperature, wind speed, noise levels, PM_2_._5_, and PM_10_, but higher relative humidity and negative air ion concentration, compared with the urban environment. These systematic contrasts demonstrated the ecological representativeness of the selected sites and validated their experimental suitability for controlled habitat comparisons.

**TABLE 3:** The differences of monitoring data between forest and urban environment.

Monitoring index	Forest environment	Urban environment	*P*
Temperature/°C	16.06 ± 0.88	16.64 ± 0.81	
Relative humidity/%	61.90 ± 4.77	55.18 ± 3.81	*
Wind speed/m/s	0	1.32 ± 0.38	**
Negative air ion concentration/ion⋅cm^–3^	835.00 ± 76.12	88.40 ± 6.43	**
Noise levels/dB	45.40 ± 3.61	62.50 ± 9.59	**
PM_2.5_/μg⋅m^–3^	21.85 ± 0.84	39.16 ± 0.82	**
PM_10_/μg⋅m^–3^	67.58 ± 1.37	153.52 ± 11.37	**

Significant difference of monitoring data between forest and urban environment were determined by One-way analysis of variance (**P < 0.01, *P < 0.05).

#### 3.2.2 Assessment of restorative environmental characteristics

Quantitative assessment using the restoration environment scale demonstrated higher restorative characteristics scores in the forest environment compared with the urban environment across all dimensions (being away, extent, fascination, and compatibility), with particularly notable differences in extent (*P* < 0.01; Table 4). These findings empirically validate the superior restorative potential of forests for psychological recovery.

**TABLE 4:** The differences of restorative environment characteristics between forest and urban environment.

Restorative characteristics	Forest environment	Urban environment	*P*
Being away	5.38 ± 1.18	4.89 ± 1.14	
Extent	6.15 ± 0.71	5.07 ± 0.98	**
Fascination	5.30 ± 1.09	4.89 ± 1.29	
Compatibility	5.17 ± 0.66	4.56 ± 0.93	

Significant difference of restorative characteristics between forest and urban environment were determined by One-way analysis of variance (**P < 0.01).

### 3.3 Emotional states

#### 3.3.1 POMS scores before and after experience

**FIGURE 3:**
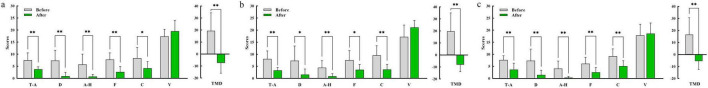
The difference of POMS scores before and after experience. **(a)** Therapist-guided forest healing. **(b)** Unguided forest exposure. **(c)** Urban exposure. **P < 0.01, *P < 0.05.

The analysis of emotional states before and after experience revealed significant improvements across all experimental groups (Figure 3). The therapist-guided forest healing group demonstrated statistically significant reductions in negative affect scores: Tension-anxiety, depression-dejection, anger-hostility, fatigue, and TMD were at the 0.01 level, with confusion showing significant reduction at the 0.05 level. Vigor scores exhibited a nominal increase, but the difference failed to attain statistical significance. The unguided forest exposure group exhibited similar patterns with significant decreases in tension-anxiety, anger-hostility, fatigue, confusion, and TMD at the 0.01 level, whereas depression-dejection decreased at the 0.05 level. The vigor scores also showed an increasing trend, although the change did not reach statistical significance. Even the urban exposure group exhibited significant reductions across all negative dimensions: tension-anxiety, depression-dejection, anger-hostility, fatigue, confusion, and TMD. The increase in vigor scores was not statistically significant.

These consistent findings across three groups suggest that all the outdoor experiences may effectively enhance emotional regulation, particularly in reducing negative affect states, regardless of setting characteristics and experience modalities.

#### 3.3.2 Emotion regulation benefits among the groups

An analysis of covariance was conducted on pre-post score changes to assess between-group differences in mood regulation efficacy (Table 5). The results indicated non-significant between-group differences in overall emotion regulation effects (*P* > 0.05). However, given the relatively small sample size of this pilot study, relying solely on *P*-values may increase the risk of Type II errors. Therefore, Cohen’s *d* was calculated to evaluate the magnitude of the intervention effect (Effect Size).

**TABLE 5:** The differences of emotion regulation benefits among the groups.

Mood states	Therapist-guided forest healing	Unguided forest exposure	Urban exposure
Δ (Tension-Anxiety)	3.75 ± 3.15	4.67 ± 3.43	4.00 ± 3.08
Δ (Depression-dejection)	6.50 ± 3.74	5.78 ± 6.80	5.89 ± 4.51
Δ (Anger-hostility)	5.00 ± 3.63	3.56 ± 3.05	3.78 ± 2.99
Δ (Fatigue)	5.13 ± 2.80	4.00 ± 2.18	3.56 ± 3.94
Δ (Confusion)	4.25 ± 4.17	5.89 ± 3.44	4.11 ± 2.37
Δ (Vigor)	-2.13 ± 5.00	-3.89 ± 3.26	-0.67 ± 4.61
Δ (The total mood disturbance)	26.75 ± 11.90	27.78 ± 13.89	22.00 ± 11.94

Analysis revealed that although the difference in TMD reduction did not reach statistical significance, the unguided forest exposure group exhibited a approaching medium effect size compared to the urban exposure group (*d* = 0.45). Similarly, the therapist-guided group showed a small-to-medium effect size over the urban group (*d* = 0.40). Notably, the effect size between the therapist-guided and unguided forest groups was negligible (*d* = 0.08), suggesting that the presence of a therapist did not substantially amplify the benefits of short-term forest bathing in this context.

Overall, both forest groups exhibited numerically greater improvement compared with the urban exposure group. These effect size estimates suggest that the lack of statistical significance may be attributable to limited statistical power, and the intervention likely holds practical utility for stress reduction.

### 3.4 Correlation between emotion regulation benefits and restorative environmental characteristics

**FIGURE 4:**
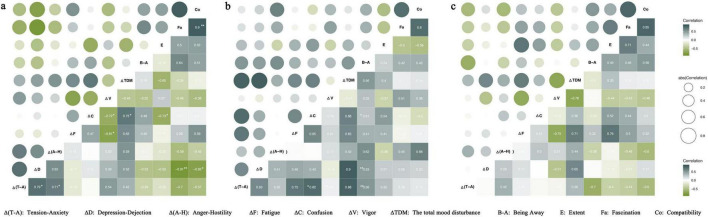
Correlation heatmap between emotion regulation benefits and restorative environmental characteristics. **(a)** Therapist-guided forest healing. **(b)** Unguided forest exposure. **(c)** Urban exposure. **P < 0.01, *P < 0.05.

We conducted correlation analyses between subjective measures of emotion regulation benefits (post-intervention change scores, Δ) and restorative environmental characteristics (assessed via questionnaire) to elucidate the mechanisms underlying emotion regulation benefits (Figure 4). The results revealed distinct but inconsistent patterns across groups. In the therapist-guided forest healing group, significant negative correlations were observed between Δ (depression-dejection) and fascination (*P* < 0.01), Δ (depression-dejection) and compatibility (*P* < 0.05), and Δ (confusion) and extent (*P* < 0.05) (Figure 4a). In the unguided forest exposure group, Δ (anger-hostility) displayed a significant positive correlation with compatibility (*P* < 0.05) (Figure 4b). In the urban exposure group, Δ (tension-anxiety) showed a significant negative correlation with being away (*P* < 0.05), whereas Δ (fatigue) showed a significant positive correlation with extent (*P* < 0.05) (Figure 4c). No consistent directional relationship emerged across groups, suggesting that the mechanism of emotion regulation benefits still needs further exploration.

## 4 Discussion

### 4.1 Emotion regulation benefits of short-term forest healing experiences

The data from this study indicated that even a brief outdoor experience could lead to measurable improvements in emotional states, particularly in alleviating negative emotions. This demonstrated the significant emotional value of changing one’s habitual activity spaces and daily rhythms by immersing them in different environments. Previous studies also suggested that the positive psychological effects were amplified when people engaged with each other and participated in outdoor activities in novel ways (Cameron-Faulkner et al., 2018; Cooley et al., 2020; Wang et al., 2021; Mattsson et al., 2022; Fernee et al., 2024). Further analysis of the research data revealed that individuals participating in activities within forest environments exhibited greater improvements in emotional wellbeing compared with those in urban settings. This highlighted the enhanced emotion regulation benefits provided by natural forest ecosystems. Simultaneously, this study incorporated synchronized meteorological monitoring to quantitatively assess the environmental conditions at two sites (forest and urban environments) while also employing questionnaires to subjectively evaluate the restorative potential of these environments. Both subjective and objective data consistently indicated that forests possessed more environmental elements beneficial to human health, such as a more comfortable thermal environment, cleaner air, greater tranquility, and stronger restorative characteristics. From the perspective of environmental psychology, these factors may serve as a crucial environmental pathways for emotional regulation. Compared to urban environment, the forest’s restorative exposome facilitates more effective psychological adaptation, providing a material basis for achieving significant health benefits. This finding aligns with previous results (Azwardi et al., 2021; Marselle et al., 2021; Piaggio and Siikamäki, 2021; Gao et al., 2024), concluding that forest environments contain more health-promoting substances and elements, thereby providing a favorable material and environmental basis for achieving health benefits.

However, the research data do not support the conclusion that the forest healing guided by a forest therapist yield significantly greater health benefits compared with free walking in forests. This may suggest that for short-term (3-h) interventions, professional therapist-guided forest healing might not necessarily exert significantly enhanced emotional regulation effects compared with unguided forest exposure within this specific sample. These findings should be regarded as preliminary, indicating the role of forest therapists warrants further investigation, particularly regarding the dose (e.g., 2 h, 5 h, 10 h, 2 days, etc.) of healing exposure required for measurable impacts (Hsieh and Li, 2022). On the contrary, studies indicate that the spatiotemporal distribution of health-promoting elements in forests is associated with the physiological cycles of plants (Damasceno et al., 2024). For instance, phytoncides, which are volatile organic compounds emitted as part of plant defense mechanisms (Jing et al., 2024; Kheam et al., 2024; Tanarsuwongkul et al., 2024), enhance immune function and reduce stress in humans (Li, 2022). Meanwhile, monitoring data reveal significant seasonal and diurnal variations in human thermal comfort and negative ion concentrations (Wang et al., 2020; Zhang et al., 2021). In other words, the timing of forest activities influences the intensity of exposure to beneficial factors, thus potentially modulating physiological responses. Thus, optimizing the scheduling of forest healing experiences may require further analysis of real-time environmental monitoring data.

### 4.2 Cost-effectiveness and public health implications of unguided forest exposure

Contrary to our initial hypothesis, the therapist-guided forest healing sessions did not yield statistically superior emotional regulation benefits compared to unguided forest exposure (*d* < 0.2). However, from the perspective of environmental psychology and public health, this “non-significant difference” is a highly positive finding regarding cost-effectiveness. It suggests that the therapeutic potential of the forest environment itself is robust enough to elicit significant mental health benefits without the need for immediate professional intervention. This finding has profound implications for policy and resource allocation. Professional forest therapy requires trained personnel and structured organization, which can limit accessibility and scalability. In contrast, unguided forest bathing is a low-cost, easily accessible self-care strategy. Our results indicate that simply providing accessible green spaces and encouraging “nature prescriptions” can be a sufficient measure for daily stress management among graduate students. This supports a scalable public health model where expensive professional resources are reserved for clinical populations, while unguided nature exposure serves as a primary preventative strategy for the general public.

### 4.3 Limitations

Despite the significant emotional benefits observed, several limitations warrant caution in generalizing these findings. First, the study’s external validity is constrained by its homogeneous sample, small size and consisting of graduate students from a single academic institution in Beijing with a predominant representation of female participants. Given that gender-based differences and cultural backgrounds can influence emotional regulation and environmental perception, the findings may not fully extend to other populations. Second, the environmental variability of the study site (Xishan National Forest Park) represents a specific ecological context; thus, the restorative outcomes might differ in diverse forest types or geographical regions. Third, this study employed a cross-sectional design measuring only acute responses immediately after the intervention. The durability of the emotional benefits remains unknown. Future research should incorporate longitudinal follow-ups (e.g., 24 h, 1 week post-intervention) to evaluate the sustained impact of short-term forest bathing on graduate students’ chronic stress levels.

## 5 Conclusion

This pilot study provides preliminary evidence that short-term forest bathing is a highly effective stress management strategy for graduate students, a population facing a significant mental health crisis. Our results indicated that: (1) Brief breaks in outdoor environments, whether urban or forest, resulted in measurable reductions in negative affect (tension, depression, anger, fatigue, confusion). (2) Compared to the urban environment, the forest environment’s superior restorative efficacyronment, the forest environmenton)tive affecton facinoncentrations, lower noise levels, and reduced particulate matterronmentoas a tangible physical basis for emotion regulation. (3) Crucially, this study found no statistically significant added value of therapist-guided interventions over unguided forest exposure for short-term emotion regulation. These findings support a scalable public health model: promoting low-cost, unguided nature exposure is a viable solution for mitigating mental health crises in higher education. As a preliminary trial, the non-significant statistical differences between these modalities mean these results are indicative rather than definitive. Future research requires large-scale Randomized Controlled Trials (RCTs) to validate these findings and assess the durability of these emotional benefits.

## Data Availability

The datasets presented in this article are not readily available because. Requests to access the datasets should be directed to zhangzhiyong@caf.ac.cn.
